# Antiseptics on the Battlefield

**Published:** 1901-12-07

**Authors:** 


### Hospital Clinics and Medical Progress.

### ANTISEPTICS ON THE BATTLEFIELD.

Recognising as we all do the importance of asepsis in surgical work, and fully impressed as we all are with the fact that the magnificent results of modern surgery are due to the success of modern methods in preventing the septic infection of wounds, it was not perhaps to be wondered at that when tales came pouring in from the seat of war as to the wonderful recoveries made by our soldiers from injuries which used to be considered as necessarily fatal, we should somewhat plume ourselves in regard to the benefits derived from the use of antiseptics in military surgery. Such being the case, the statements made by Mr. Watson Cheyne [1] as to the degree of asepsis really attained "at the front" are as interesting from a surgical point of view as they are disappointing to those who have attributed so much to antiseptics. He deals, of course, only with what happened in the treatment of patients in the field. When they got to the base the value of recent advances in surgery at once came into play, and from that period onward the cases did incomparably better than they would have done before antiseptics were introduced. But in the front he thinks that modern surgery and active asepsis had very little to do with the results, which in his opinion are mainly attributable to the character of modern bullet wounds, and to the climate in which these wounds were inflicted. In fact, he goes so far as to say that if these wounds had been made with the old round bullet in a damper climate (say in the Crimea) the immediate results under the methods of treatment adopted in the field would not have been so very much better than those formerly obtained, in spite of the recent advances in surgery and the introduction of Listerism, except so far as this, that whereas in former days wounds were meddled with, probed, opened up, poulticed, and so forth, in the present campaign they were left alone and mischievous interference was avoided. But even this non-interference Mr. Watson Cheyne attributes not altogether to the teachings of modern surgery, but largely to the fact that from the large number of wounds which occurred in the early battles, and from the small *personnel*, there was difficulty in finding time to open up all these wounds. After describing in a very convincing fashion the position of a soldier lying wounded in the field, and the necessarily soiled condition of the "field dressing" before it gets applied to the wound, a dressing which even to begin with, and before it has been soiled by the soldier's hands, "is more a sort of clean rag than any actively antiseptic dressing," he goes on to say that when the patient at last arrived at the dressing station, and even in cases where it was essential that the wound should be redressed, practically little or nothing was done in the way of thorough disinfection, of course owing to the circumstances of the case. A few men seemed to make a fairly efficient attempt to disinfect the wounds as soon as they came under their care, but in the majority of cases the lotions at hand were quite insufficient and inefficient, "and practically no real disinfection was carried out." Yet, he says, it is self-evident that the results which were met with could not have been attained unless the wounds had in many cases remained aseptic. "The point is that this asepsis was not brought about by the surgeon, but depended on the climate and the nature of the bullet wound." It must, not, however, be supposed that bullet wounds invariably escaped sepsis. Even small wounds made by rapidly-travelling bullets often had suppuration along the track, while in cases where the wound was an inch in size, or larger, "sepsis invariably occurred." Cases also were met with in which extremely rapid acute septicæmia and emphysematous gangrene occurred, as well as many cases of prolonged suppuration and osteomyelitis, some of which were only rescued by early and high amputation. Undoubtedly the nature of the wound was much in favour of the wounded in many cases, but quite as important as the size and nature of the wound were the conditions under which it was inflicted, and especially the fact that from the dryness of the South African climate the blood quickly dried up and formed a scab, the bleeding being generally quite slight in the small wounds which were so common. "The antiseptic dressing and the surgical treatment had little or nothing to do with the result in these cases, and the only way in which we can bring in modern surgery as of value is because it has taught us the meaning and value of healing under a scab." The importance of the formation of a scab is shown by the fact that when, as was the case at first, the soldiers applied the mackintosh which was included in the "field dressing" thus preventing the formation of a scab, sepsis often occurred. The gauze and the discharge when thus confined under the mackintosh were often found to be stinking within a few hours of the infliction of the wound. It was the scabbing, and not the antiseptic contained in the dressing, that was the important thing in preventing sepsis. Where the wound was too large or the discharge too great to allow the formation of a crust sepsis was almost certain to occur; "indeed," says Mr. Watson Cheyne, "I have never seen a wound of any large zize which was not septic." The question is, then, what should be done to remedy this very serious condition of affairs? It is quite clear that in no case can we hope to obtain asepsis from the first. For many hours, sometimes, it is impossible to get at the wounded; sufficient water cannot be carried on to the field even to satisfy thirst, and the doctor quickly becomes as dirty as his surroundings. The only thing that appears possible would seem to be to devise a first dressing which shall as far as possible promote the formation of an aseptic scab. The immediate and perhaps repeated dusting of the wound with some antiseptic powder would then be the first thing. Then it would seem essential that the field hospitals and dressing stations should be prepared beforehand with sterilised water and with the necessary lotions. Although in the small wounds it may be best not to disturb the crusts, in larger wounds the only chance of avoiding sepsis is by thorough disinfection of the skin and wound as soon as possible, and to do this is out of the question without proper solutions. "For a field hospital to go into action without lotions, without water, without the means of boiling water, or without proper means of illumination, is just as bad as for guns to go into action without proper fuses for the shells. These things are absolutely necessary equipments for the work, and if additional transport is required for them it must be provided." Mr. Watson Cheyne is evidently very strongly impressed with the dangers and the horrors which of necessity accompany all attempts to remove seriously wounded soldiers for any distance after an engagement, and he thinks that the ideal plan would be to arrange for the field hospitals to be left on the field of battle until it was safe for them to be transported to the base. He thinks some arrangement might be come to among the nations in order that this might be done. In the meantime what is wanted is better arrangements to prevent sepsis in the first instance, proper supplies of lotions and boiled water, proper illumination (for the operative work after a battle has to be done by night), and an increase in the *personnel.*

[1] Birmingham Medical Review, November, 1901.

